# Longitudinal study: understanding the lived experience of couples across the trajectory of dementia

**DOI:** 10.1186/s12877-021-02503-4

**Published:** 2021-10-15

**Authors:** Mary S. Mittelman, Maureen K. O’Connor, Tiffany Donley, Cynthia Epstein-Smith, Andrew Nguyen, Roscoe Nicholson, Rebecca Salant, Steven D. Shirk, Elizabeth Stevenson

**Affiliations:** 1 Department of Psychiatry, New York University School of Medicine, New York, USA; 2 Department of Neurology, Boston University School of Medicine, Boston, MA USA; 3 Department of Population Health, New York University School of Medicine, New York, USA; 4 Department of Human Development, University of Chicago, Chicago, IL USA; 5 Department of Psychiatry and Population and Quantitative Health Sciences, University of Massachusetts Medical School, Worcester, MA USA

**Keywords:** Alzheimer’s, Dementia, Couples, Relationships, Older adults, Psychosocial factors

## Abstract

**Background:**

The longitudinal study, “Couples Lived Experiences,” focuses on whether and how relationship characteristics of older couples change with the cognitive decline of one member of the couple, and how these changes affect each individual’s emotional and physical health outcomes. Until now, most psychosocial research in dementia has focused either on the person with dementia (PWD) or the caregiver separately. The previous literature examining relationship characteristics and their role in outcomes for the caregiver and PWD is scant and suffers from methodological issues that limit the understanding of which relationship characteristics most influence outcomes for caregivers and care-receivers and what other factors may mitigate or exacerbate their effects.

**Methods:**

We will enroll 300 dyads and collect information via online interviews of each member of the couple, every 6 months for 3 years. Relationship characteristics will be measured with a set of short, well-validated, and reliable self-report measures, plus the newly developed “Partnership Approach Questionnaire.” Outcomes include global quality of life, subjective physical health, mental health (depression and anxiety), and status change (transitions in levels of care; i.e., placement in a nursing home). Longitudinal data will be used to investigate how relationship characteristics are affected by cognitive, functional, and behavioral changes, and the impact of these changes on health outcomes. Qualitative data will also be collected to enrich the interpretation of results of quantitative analyses.

**Discussion:**

Psychosocial interventions have demonstrated effectiveness in promoting the wellbeing of PWD and their caregivers. The knowledge gained from this study can lead to the development or enhancement of targeted interventions for older couples that consider the impact of cognitive and functional decline on the relationship between members of a couple and thereby improve their wellbeing.

**Trial registration:**

This study has been registered with ClinicalTrials.gov. ClinicalTrials.gov Identifier is: NCT04863495.

## Background

As the population continues to age, there is a corresponding increase in the number of people with Alzheimer’s disease and related dementias (ADRD) [1, 2] and the number of family caregivers. While funding for the development of novel pharmacological and disease-modifying targets often takes center stage [3, 4], to date, none has been identified that can substantially modify the disease course. Meanwhile, the financial and emotional cost to patients and families and the cost to the federal health care budget continue to grow.

Psychosocial interventions have substantial evidence of efficacy in improving outcomes for caregivers and persons with dementia (PWD) (e.g., the NYU Caregiver Intervention (NYUCI) [5–8]. The health outcomes demonstrated by the NYUCI are largely mediated by social support [9]; Moreover, many large-scale epidemiologic studies corroborate this finding and demonstrate that social support can predict better health outcomes and reduce the risk for premature mortality [10].

Partner relationships may be a particularly important form of social support, which is why the current study focuses on relationship characteristics of couples. In facing a chronic illness, both partners need support, and each is generally the primary support of the other [11]. Spouses frequently share stressors appraising them as “ours” rather than “mine”, pool resources, and actively engage in joint coping efforts [12]. Cross-sectional and longitudinal studies have documented concordance in levels and changes in spouses’ mental health and wellbeing. For example, in a longitudinal analysis of a repeated household survey, one partner’s mental health status predicted about 25% of the variance in the other partner’s mental health [13]. Older adults, whose relationships are generally of greater duration, were likely to have a higher concordance, which may reflect dynamic processes and shared experiences that increase with the length of the marriage [14]. The burden of dementia on older couples is especially poignant, and although they generally endure the illness together, cognitive decline and behavioral symptoms alter the role of the partner-caregiver and impact their relationship in ways this study aims to elucidate.

The stress process model [15] suggests that caregiver wellbeing is affected not only by primary stressors originating directly from illness and care of the patient but also from secondary stressors (such as family conflict and constriction of social activities). Social support and appraisal of the meaning of the stressors can mediate the effects of primary caregiving stressors on caregiver wellbeing [16]. In the early 1990s, the growing awareness of the impact of illnesses such as ADRD on the quality of the marital relationship led to an extension of the stress process paradigm to include a concept that was defined as dyadic coping, a process which includes the stress signals of one partner, the perception of the signals by the other partner, and the reaction of the other partner [17]. This extended version of the stress process paradigm provides a conceptual motivation for our choice of measures.

The primary goal of the current study is to further our understanding of the impact of relationship characteristics on outcomes for the caregiver and care-recipient in spousal or partner dyads affected by cognitive decline. This goal will be pursued through three specific aims: 1) to determine the cross-sectional association between dyadic relationships and health outcomes; 2) to evaluate the change in the couple’s relationship over time, the potential causative factors, and the implications this change holds for the wellbeing of each member of the couple, and 3) to use qualitative data collection and analysis to further explore the personal impact of changes in the relationship and enhance the interpretation of the results of the quantitative analyses.

### Limitations to prior research

The body of literature examining relationship characteristics and their role in outcomes for the caregiver and care-recipient is scant and suffers from methodological issues that limit our ability to understand which relationship characteristics most influence outcomes for caregivers and care-recipients and what other factors may mitigate or exacerbate their effects. Although many studies look at relationship characteristics and their association with outcomes for caregivers and PWD cross-sectionally, we are not aware of any study that has examined changes in multiple aspects of relationship characteristics that occur over the course of the illness and how those changes impact health outcomes. Further, most of the studies that explore relationship characteristics do so only from the caregiver’s perspective [18], or rely on ratings made on behalf of the PWD by the caregiver, another family member, or friend, as once he or she has dementia, even in the early stage, the PWD had been viewed as no longer being a reliable reporter [18, 19]. Our review of the literature suggests that other limitations to prior research on relationship characteristics and dementia outcomes include small sample sizes, and unexamined potential confounds that limit the interpretation of the importance of relationship factors.

There is a wealth of literature on the impact of caregiver factors and care-recipient factors on outcomes, mostly from the perspective of the caregiver [20]. The caregiving literature explores how factors such as social support, self-efficacy, and positive aspects of caregiving impact mental and physical health outcomes for caregivers, and transitions to residential care. The literature is also robust when considering how outcomes are affected by care recipient factors such as disease characteristics (cognitive and functional decline, neuropsychiatric symptoms). However, most clinicians would agree that the relationship itself, as separate from factors specific to each individual in the dyad, has the potential to contribute to outcomes.

### Rationale for this study

A longitudinal study of dyads living with dementia is essential to understanding how relationship characteristics change over time and contribute to health outcomes for caregivers and care-recipients. In this study, we will focus on relationship characteristics and their effect on mental and emotional health outcomes for each individual in the dyad. The dyadic nature of this study will yield information about the relationship from the perspective of both members of the dyad. The primary goal is to understand the relationship between the members of a couple over time, how it changes as one becomes cognitively, functionally, and behaviorally impaired, and how the changes in their relationship affect the wellbeing of each of them. We will assess *both* members of the dyad at varying stages of the disease, from normal through early dementia to get information on their relationship, and how it changes from *both* their perspectives.

By collecting longitudinal data on caregiver factors, care-recipient factors and relationship factors, we will be able to determine how relationship factors contribute to the caregiver and care-recipient outcomes above and beyond the well-studied individual factors. We will investigate how relationship characteristics change over time as cognitive, functional, and behavioral problems increase, and the impact of these changes on outcomes such as global quality of life, subjective physical health, mental health (depression), and the amount of time to care transitions (for example, placement in a nursing home). Qualitative data collection and analysis will be used to further explore the dynamic nature of the relationship characteristics of interest and enhance the interpretation of the results of quantitative analyses.

## Methods

### Overview

We will enroll a total of 300 couples over an 18-month period, recruited from two locations (the New York City, New York metropolitan area and the Boston, Massachusetts metropolitan area), with 150 dyads per site. Among these couples we will recruit 60 dyads in which both members have normal cognition, 140 in which one member has Mild Cognitive Impairment (MCI), and 100 in which one member has ADRD in the early stages. Information about the study will be distributed through channels such as memory clinics, trial registries and community organizations that serve older adults. While we had originally planned to conduct the assessments in person, the exigencies of the COVID pandemic necessitated changing the protocol so that all interviews will be conducted via video conferencing.

Both members of the dyad will be asked to complete an eligibility screen and provide consent to participate via an online portal. Structured self-report instruments will be administered at baseline and at 6-month intervals over a 3-year period to obtain each of their perspectives on changes in multiple aspects of their relationship. We will characterize participants throughout the study at each time point into one of three groups (normal, MCI, and ADRD) based on cognitive performance using the MoCA [21] combined with activities of daily living assessment scores using the FAQ [22] to capture decline from normal to MCI to ADRD. The assessment will be completed by both members of the dyad from normal through early dementia. If at the conclusion of a follow-up interview the participant is deemed to no longer have capacity, the data from that assessment will be excluded from analyses and the participant will not be included in further assessments. We will continue to evaluate the relationship from the caregiver’s perspective when a capacity evaluation determines that the person with dementia can no longer be a reliable respondent. If one member of a couple dies, the other member will be asked to complete an exit bereavement assessment 6 months after the death.

The baseline assessment includes measures of relationship style and potential outcomes such as depression and physical health. We will use these cross-sectional data to develop an initial statistical model which will include exogenous factors, primary stressors, mediators and outcomes (Aim 1) which can be tested in longitudinal analysis. The longitudinal study will enable us to assess the validity of this model and modify it to reflect the additional information about changes in relationships over time in response to cognitive decline in one member of the couple and the impact of these changes on health outcomes of both members of the couple (Aim 2).

**Fig. 1:**
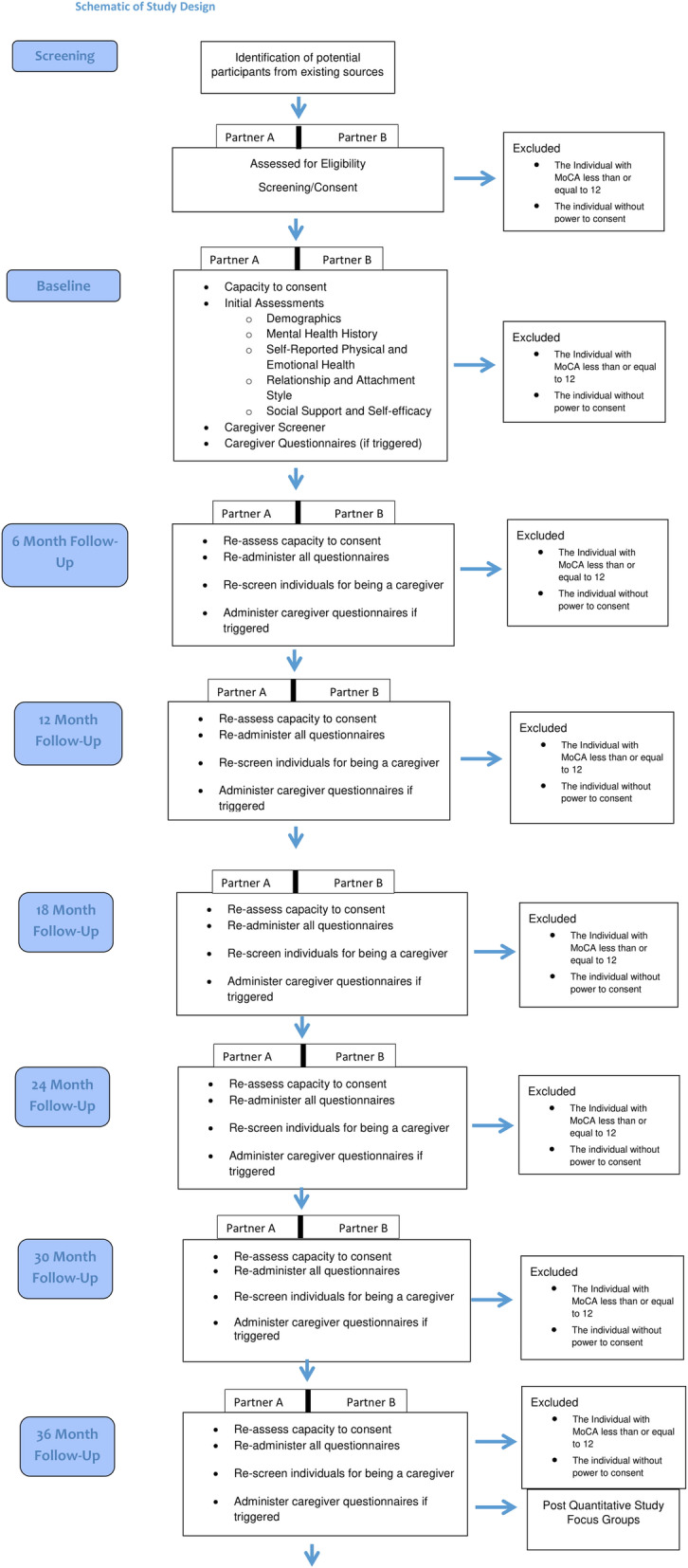
Schematic of Study Design

In addition to the quantitative study, we will hold focus groups with 32 of these couples (16 per site) after the last 6-month follow-up (3 years after the baseline assessment). A qualitative examination using the results of these focus groups will complement the longitudinal analysis by illuminating interpersonal processes by which change or continuity in relationship and couples’ wellbeing occurs (Aim 3). Qualitative analysis will also provide an understanding of the relevance of the constructs identified via the longitudinal analysis as they apply to individual members of dyads’ experience of their relationship as it was and as it is now. Figure 1 contains a schematic of the above delineated protocol.

Scientific rigor will characterize the analysis of all quantitative and qualitative data. Assessments and focus groups will be implemented by trained research personnel to assure that responses are acquired consistently and will utilize written materials that can be used by future researchers. To ensure reproducibility, we will use sensitive and reliable measures and recruit a large and diverse sample that will provide sufficient statistical power to be likely to reflect a representative sample of couples coping with aging and cognitive decline.

### Eligibility requirements

To be eligible, participants must be spouses or partners who are living together in the community at the time of enrollment. Both must be willing to participate in the intake and follow-up assessments. They must be English-speaking adults aged 65 years or older. All participants must have internet access and a device that is compatible with a HIPPA-compliant video teleconferencing application in order to be able to participate in the interviews that will be conducted via videoconference. All participants must be able to complete the informed consent process. The study will exclude anyone who is deemed not to have capacity to consent, those with a prior diagnosis of schizophrenia or other psychotic disorder or with a prior diagnosis of a chronic disabling medical condition that would make it impossible to participate in virtual study visits. Those who participate in the focus groups must be willing to have their comments recorded.

### Recruitment

Participants will be recruited from the NIH-funded Alzheimer’s Disease Research Center (M. Mittelman, Psychosocial Core Leader), the New York State-funded NYU Alzheimer’s Disease and Related Dementias Family Support Program (M. Mittelman, Director), the Boston University Alzheimer’s Disease Research Center (M. O’Connor, Research Education Core Leader), The Bedford VA Hospital Memory Diagnostic Clinic (M. O’Connor, Director), and the Alzheimer’s Family Support Center, Cape Cod, MA as well as from registers of people interested in participating in research about aging and cognitive decline such as the Banner Health Alzheimer’s Prevention Registry. These facilities collectively serve more than 1000 participants age 65 and older each year. Additional participants will be recruited via newspaper ads and social media. Efforts will be made to ensure enrollment of participants from a wide range of socioeconomic and ethnic backgrounds.

The research coordinators will answer calls about the study and administer a structured screening questionnaire virtually via a HIPPA-compliant videoconferencing platform that will include background information and initial eligibility criteria. Prospective participants, who appear to be eligible based upon the initial screen, will be invited to join the study, asked to sign an electronic informed consent and then will be scheduled for a virtual intake assessment. At baseline, the interviewer will review the protocol with the potential participants and answer any questions. Our experience in prior studies suggests that recruitment and retention will be enhanced by giving participants a sense that their efforts are valued. To do so, we will provide a small monetary compensation for completing the assessments. We will invite all participants to an annual colloquium which can be attended in person or via live streaming, at which time we will share information on the progress of the study. Participants will also be offered individual feedback in an interactive session with a study clinician after their last assessment; they will receive a written summary of their personal and relationship characteristics, based on their responses to our assessment.

### Assessment

#### Process

Assessments will be conducted via videoconferencing, using a HIPPA-compliant video-conferencing platform. Each participant will complete the assessments separately from his or her partner. The assessments will be administered by a research coordinator verbally, with visual cues shared on screen for certain instruments when necessary. The assessment process will be consistent and standardized at both sites and for all interviewers. We anticipate that it will take couples an average of 2 h at baseline and 1.5 h at follow-up to complete the assessments, based on the results of a pilot of the battery with eight older individuals who completed it in as little as 40 min and no more than 1 h.

We will collect data from each member of the couple that includes demographic characteristics, psychosocial attributes (e.g., perceived social support, self-efficacy), relationship factors (relationship satisfaction, relationship continuity, partnership approach) and outcomes, including global quality of life, physical health, mental health (depression and anxiety) and status change (for example, placement outside the home). This assessment battery will enable us to estimate the contribution of relationship factors, above and beyond the commonly well-studied individual factors, to caregiver and care-recipient outcomes.

For couples in which one participant identifies as the caregiver of the other, we will use additional instruments to measure caregiver responses to the effects of cognitive changes (e.g., loss of intimate exchange, ability to identify positive aspects of caregiving) and care-recipient attributes (i.e., functional ability and behavioral problems from the perspective of the caregiver). It should be noted that, if one member of the couple declines in cognitive function and the other doesn’t identify as a caregiver, the caregiver battery will not be used.

At each follow-up visit, the same procedure used during the intake visit will be followed. As the individuals with cognitive impairment or ADRD progress in the severity of their symptoms, they will continue in the study until scoring 12 or less on the MoCA or are deemed to lack capacity, after which they will be withdrawn from the study; their study partner will continue to participate.

If scores on any of the measures in the assessment indicate a participant may have had a significant change in cognition or emotional status, we will notify both members of the dyad, and we will provide the opportunity to discuss these results with a study clinician and receive appropriate referrals.

#### Assessment battery

The measures selected for this study were chosen to enable us to fulfill our Specific Aims. Many of these scales are included in the NIH toolbox.

All study participants will complete the following assessment battery at baseline:

Demographic questions, including age, gender, race/ethnicity, socio-economic status, characteristics of marriage (years married, first marriage?), number of children, work history (ever worked, currently working). Demographic questions will be administered only at baseline.

Cultural expectations of marriage (traditional/nontraditional) –One item: “How traditional do you consider your marriage, on a scale of 0 to 10, where 0 is nontraditional and 10 is very traditional?”

Attachment Questionnaire [23]: This 3-item questionnaire focusing on three distinct attachment styles; secure attachment, Anxious/Ambivalent attachment, and Avoidant attachment each rated on a 7-point Likert scale with “1= Strongly disagree” and “7= Strongly agree”. The Attachment Questionnaire will only be administered at baseline, as it is considered to be a stable attribute for the purpose of this study.

Revised Dyadic Adjustment Scale [24]: This 14-item scale designed to measure relationship satisfaction in three domains: 1) Dyadic Consensus, 2) Dyadic Satisfaction, and 3) Dyadic Cohesion.

Partnership Approach Questionnaire [25]: This 28-item scale assesses respondent’s caregiving style; whether the caregiver employs an individual-based caregiving style or views the couple as a team working towards the caregiving needs placed upon them. Rated on a 7-point Likert scale with “1= completely disagree” and “7= completely agree”.

Measures of family style (SCORE) [26]: This 15-item questionnaire measures the quality of the respondent’s family life. Rated on a 5-point Likert Scale with “1= describes us: very well” and “5= describes us: not at all”.

Lubben Social Network Scale-6-Item Version [27]: This 6-item questionnaire measures the number of family members and friends they hear from once a month, free close to, and feel at ease with discussing private matters, to get an overall understanding of their social support network.

Social Support availability [28]: This measure includes three 4-item subscales of emotional, instrumental and informational support.

Loneliness [29]: Measures feelings of loneliness, whether or not one has a large social network.

Life Orientation [30]: This 10-item assessment of feelings of generalized optimism vs pessimism, rated on a 5-point scale ranging from “I agree a lot” to “I disagree a lot”.

Meaning and Purpose in Life [31]: This 18-item measure of life purpose rated on a 5-point Likert scale from Strongly Disagree to Strongly Agree.

Coping self-efficacy [32]: This 10-item questionnaire assessing feeling of self-efficacy in daily activities, rated on a 5-point Likert scale with “1 = Never” and “5= Very Often”.

Geriatric Depression Scale [33]: This 15-item measure of symptoms of depression among older adults; 15 ‘yes’ or ‘no’ questions in which participants respond in reference to how they felt over the past week.

Beck Anxiety Inventory (BAI) [34]: This 21-item measure of symptoms of anxiety during the past month on a 4-point scale from “Not at all” to “Severely-it bothered me a lot.”

Physical Health Questionnaire [35]: This measure includes three global self-rated physical health items from the OARS battery of questionnaires (ICC = 0.83) to assess subjective evaluation of health The sum of the 3 questions is used to measure SRH.

Sleep Survey: This measure includes two items to assess subjective sleep quality over the previous month.

EuroQol Questionnaire Global Quality of Life [36]: This measure assesses overall quality of life on a scale of 0–100 with “100 being the best quality of life imaginable”.

Caregiver Screener: Once all of the above questionnaires are completed, all individuals will be administered a short Caregiver Screener which asks if individuals identify as a caregiver, if their care partner needs additional help with tasks, how often they provide care, and utilization of outside resources to assist in caring for their partner. The screener determines whether additional questionnaires should be implemented with the caregiving partner.

*Functional Activities Questionnaire [22]: This 10-item measure to assess the abilities of the person with dementia. Each functional activity assessed as: (3) Dependent; (2) Requires assistance; (1) Has difficulty but does by self; (0) Normal or (0) Never did [the activity] but could do now; (1) Never did and would have difficulty now.

*Revised Memory and Behavior Checklist [37]: This 24-item measure assesses the functioning and behavior of an individual with dementia. Caregivers are asked if a certain behavior is present and if so how much it bothers them, on a scale from “Not at all” to “Extremely.”

*Service Utilization: Scale about utilization of different support services, including respite care, support groups, food services, etc.

*Birmingham Relationship Continuity Measure [38]: This 23-item scale assessing caregiver’s appraisal of relationship continuity, their negative and positive emotional reactions to the caregiving role; rated on a 5-point Likert scale ranging from “Agree a lot” to “Disagree a lot”.

*Deprivation of Intimate Exchange [15]: How much have you lost?: (a) Being able to confide in your (relative); (b) The person that you used to know; (c) having someone who really knew you well. Response categories: (4) completely; (3) quite a bit; (2) somewhat; (1) not at all.

*Positive Aspects of Caregiving Scale [39]: This 9-item assess caregivers’ perceptions regarding the positive aspects of caregiving; two components, (1) self-affirmation and (2) outlook on life rated on a 1–5 Likert scale with “1= disagree a lot” and “5= agree a lot” .

Montreal Cognitive Assessment (MoCA) [21] will be administered to all participants at the end of each interview. The MoCA is a screening instrument that assesses multiple cognitive domains: attention and concentration, executive functions, memory, language, visuospatial skills, conceptual thinking, calculations, and orientation.

Status Change Form, indicating the type and date of transitions, such as residential care placement, illness, move, refusal and death will be filled out when the participant informs the study personnel of the change, which may be during an ad hoc call, a scheduling call, or a follow-up assessment.

The questionnaires listed above, except for demographics, cultural expectations of marriage, and the Attachment Questionnaire, will be administered at all of the follow-up visits, unless a change in living arrangements or death of one member of the couple occurs, when the procedure will be altered to suit the new circumstances:

1. Change in Living Arrangements: If the living arrangements for the couple change during study participation, adjustments will be made to the assessment schedule to adjust for the changes in interaction and caregiving responsibilities. Visits will continue at regular intervals for both individuals if cognitively capable of consenting. All measures, except for the Revised Memory and Behavior Checklist (if caregiver), will be administered.
2. Bereavement: Should one member of the couple die, the other individual will be asked to complete an exit bereavement assessment 6 months after the date of death, which will include the following measures from the main assessment battery: Dyadic Adjustment Scale, SCORE Measures of Family Style, Lubben Social Network Scale, Social Support Availability, Loneliness, Life Orientation, Meaning & Purpose in Life, Coping Self-Efficacy, Geriatric Depression Scale, Suicidality Evaluation, Beck Anxiety Inventory, Physical Health Questionnaire, and EuroQoL. In addition to these measures, the Texas Revised Inventory for Grief [40] will be administered during this assessment.

Texas Revised Inventory of Grief-Present [40]: This 13-item measure assess an individual’s present feelings of grief, rated on a scale from “1 = completely true” to “5 = completely false”.

##### Sample size calculation

Sample size was based upon the proposed longitudinal analysis investigating the effect of clinical group and relationship characteristics on changes in the primary outcomes over time. With an initial sample size of 300 couples (600 individual participants) and a potential dropout rate of 20% per group, we have powered the analysis to provide useful outcomes with a sample size of 240 dyads [41]. We have calculated the necessary sample size to sufficiently power the analyses for the smallest clinically substantive effect on the outcomes using the Geriatric Depression Scale scores in each of our three groups (cognitively normal controls, MCI, and AD). We made the following assumptions: 1) the minimum detectable effect size is 0.20, 2) there is 1 baseline measurement and either 3 or 5 post-baseline measurements, 3) two-tailed alpha error is 0.05, 4) power was set 0.8, and 5) the within-person association for over-time measurement is 0.7. With these assumptions, the study will need 50 patients for 3 follow-up measurements and 48 patients for 5 follow-up measurements per clinical group to adequately investigate the primary outcome, Geriatric Depression Scale scores [42]. The addition of covariates as well as multiple predictor variables and attrition will necessarily reduce this power; therefore, we have increased the sample size for those groups that may suffer most from attrition and offer the most clinical relevance (MCI and AD). Also, we will use model-building techniques (e.g., backward elimination) to fit the most parsimonious models. The use of sensitive and reliable measures and employing mixed-effects models to analyze repeated measures, which preserve all available data, will also, in general, improve power [43]. Lastly, the use of continuous predictor variables when available, e.g., MoCA score should also increase power over the use of categorical variables. With a sample of 300 couples at baseline, we are well powered to conduct the proposed analyses.

##### Statistical analysis plan

Aim 1: To determine the association between dyadic relationships and health outcomes. We will conduct a cross-sectional analysis of the baseline interviews, stratified by cognitive function, across the disease spectrum from normal to dementia, assessing multiple aspects of the relationship from the perspective of each member as well as a comprehensive set of psychosocial attributes. The initial analyses for this aim will be bivariate in nature, and although largely descriptive, the results of these analyses will inform us as to what variables will be important to consider in more complex models in determining what relationship characteristic contribute to the health of individuals, both for the caregiver and PWD, across the normal-AD spectrum both cross-sectionally and longitudinally. These variables will be compared in predicting groups using multinomial logistic regression. In the case where variables are too highly correlated to be included in models together, strategies will be developed to choose the most important variables, either a priori or according to statistical criterion such as R-squares or standardized estimate comparisons, or to run multiple analyses with different sub-sets of variables.

Aim 2: To evaluate the change in the couple’s relationship, the potential causative factors that contribute to change in relationship characteristics, and the implications this change holds for the wellbeing of each member of the couple. Parameterization of repeated measures models [44]. The same fundamental procedures will be used for fitting all repeated measures models in this proposal. Because respondents will not be randomized, there may be differences across groups at baseline (e.g., age, education, relationship style). Therefore, we will include baseline measures in the trajectory analysis. Time will be treated as linear, but quadratic terms may be included if such a trend is graphically suggested. Correlation structures will be fit as autoregressive, generally the most appropriate for repeated measures data. Distributions of dependent variables will be either normal with an identity link or binary with a logit link. Models will be fit in SAS PROC GENMOD or PROC MIXED. For this aim, models will include a group by time interaction and will adjust for baseline variables. The model will be reduced in a backwards elimination fashion, removing variables that are non-significant and not important confounders. A significant interaction term in the final model will imply a significant difference in trajectories between groups, in which case adjusted mean and rates of changes will be presented separately for each time point. Post hoc analyses may involve testing pairwise comparisons across disease severity at specific time points or testing hypotheses around specific types of trajectories based upon observation of the adjusted least-square means or predicted probabilities by severity and time. Any post hoc analyses would be considered exploratory and p-values would be adjusted for multiple comparisons.

Longitudinal data will allow us to determine what relationship variables remain relatively static or are dynamic throughout the study and disease progression. This information will provide insight into what relationship characteristics may be responsive to psychosocial interventions. In addition, observing changes over time, via time-varying predictors and outcomes, will provide a closer approximation of causal inference; baseline severity along with changes in the relationship may be predictive of outcomes.

Aim 3: To use qualitative data collection and analysis to further explore the dynamic nature of the relationship characteristics of interest and enhance the interpretation of the results of the quantitative analyses. While the focus group interview guide will be heavily informed by the findings of the quantitative data, the analysis of the focus group data will be conducted according to the principles of framework analysis, which provides a systematic and transparent approach to qualitative research and can be applied deductively to answer a priori research questions and inductively to identify themes, insights or constructs. Framework analysis [45] includes five stages: familiarization; identifying a thematic framework; indexing; charting; mapping and interpretation. This analysis involves the identification and clustering of common themes in the data which will be carried out independently by two researchers. The researchers then will work together to reach a concordance of views on common themes. This independent analysis will be conducted by two trained qualitative researchers using NVivo software. As needed, a third qualitative researcher will be involved to resolve situations where concordance of themes cannot be reached.

#### Attrition and sensitivity analysis

We expect an attrition rate of approximately 20%. Attrition may be greater for some clinical groups than for others (e.g., spousal caregivers of a PWD. If that is the case, variables of interest may be associated with attrition, and missing data will not be at random. This possibility could then bias results. To address this possibility, we will test to determine whether missing data are missing at random, by investigating if baseline measures may predict attrition and employ a statistical test to determine missingness [46–48]. If missing at random is observed, then the planned analysis will be stratified accordingly.

## Discussion

The burden of ADRD on older couples is especially poignant, as they generally weather the illness together, with shrinking social networks to support them. Relationship characteristics have been shown to impact dementia outcomes for both the caregiver and the PWD. However, there are many limitations to prior research on relationship characteristics and dementia outcomes, including an almost exclusive focus on cross-sectional data collection, small sample sizes, and unexamined potential confounds that limit the interpretation of the importance of relationship factors. Longitudinal study of dyads living with ADRD is essential to understanding how relationship characteristics change over time and contribute to health outcomes for caregivers and persons with dementia. While relationship quality has an impact on both the caregiver and the care-recipient; the relationship between them from both their perspectives has rarely been studied.

The person who takes on the caregiving role may or may not consider him or herself a caregiver. This study will illuminate our understanding of when and why people identify themselves as caregivers or choose not to. Variability in interpretation of the caregiving role may be accounted for by the impact of other characteristics of each of the members of the dyad that are being investigated in this study.

Understanding the effects of dementia on couple relationships is a critical step needed to optimize existing evidence-based approaches and develop new ones for maintaining the wellbeing of caregivers and PWD. The findings of this study will inform the development of interventions to maximize the wellbeing of both members of a couple as one of them experiences cognitive decline and dementia.

## Data Availability

The investigator who proposed to use the data will have access to the data upon reasonable request. Requests should be directed to Mary.Mittelman@nyulangone.org. To gain access, data requestors will need to sign a data access agreement.

## Abbreviations

ADRD: Alzheimer’s Disease and Related Dementia
MCI: Mild Cognitive Impairment
PWD: Person with Dementia
